# Surgical readmissions: results of integrating pre‐, peri‐ and postsurgical care

**DOI:** 10.1002/nop2.52

**Published:** 2016-05-10

**Authors:** Katia Noyes, Janet Baack‐Kukreja, Edward M. Messing, Luke Schoeniger, Eva Galka, Wei Pan, Cai Xueya, Fergal J. Fleming, John RT Monson, Supriya G. Mohile, Todd Francone

**Affiliations:** ^1^ Surgical Health Outcomes & Research Enterprise (SHORE)RochesterNew YorkUSA; ^2^ Department of SurgeryUniversity of Rochester Medical CenterRochesterNew YorkUSA; ^3^ Department of UrologyUniversity of Rochester Medical CenterRochesterNew YorkUSA; ^4^ Department of Biostatics and Computational BiologyUniversity of Rochester Medical CenterRochesterNew YorkUSA; ^5^ Department of Medicine, Hematology/OncologyWilmot Cancer InstituteUniversity of Rochester Medical CenterRochesterNew YorkUSA; ^6^ Lahey Hospital & Medical CenterBurlingtonMassachusettsUSA

**Keywords:** Discharge process, integrated care, multidisciplinary care, readmission, surgical outcomes

## Abstract

**Aims:**

To explore the feasibility of recruiting surgical oncology patients and implementing a surgical integrated discharge (SID) programme led by advanced practice providers (APP).

**Background:**

Burden of illness and complexity of treatment regimen makes it challenging for surgical oncology patients to participate in research. Surgical oncology nurses may have the necessary expertise to overcome this problem.

**Design:**

Controlled longitudinal prospective observational study.

**Methods:**

The SID programme included multidisciplinary care coordination, regular communication among APPs and proactive postdischarge follow‐up. Administrative databases were used to identify matching historical controls (*n* = 113) and evaluate programme outcomes.

**Results:**

Patient enrolment was 84%. The main challenges for the programme implementation included incompatible health information systems among care settings, variation in care processes among hospital units and need for provider behaviour change.

**Conclusions:**

Most surgical oncology patients are willing to participate in outcomes programmes when contacted by familiar clinical personnel but programme implementation requires leadership support, communication among care teams and training and infrastructure.

## Introduction

Since the passage of the Patient Protection and Affordable Care Act in March 2010, the Centers for Medicare and Medicaid Services (CMS) has been authorized to penalize hospitals for unplanned readmission rates deemed to be *‘*excessive*’* (Cms.Gov 2014b). In the first year alone, CMS procured about $300 million through the application of a 1% penalty for this infraction, which increased to 3% in 2015 (Joynt & Jha 2013). While this measure is currently being applied only to discharge diagnoses of heart failure, pneumonia and myocardial infarction, it is envisaged to soon expand and encompass other clinical areas, including surgical readmissions (Brandao *et al*. 2014, Keeney *et al*. 2015a,b).

**Figure 1:**
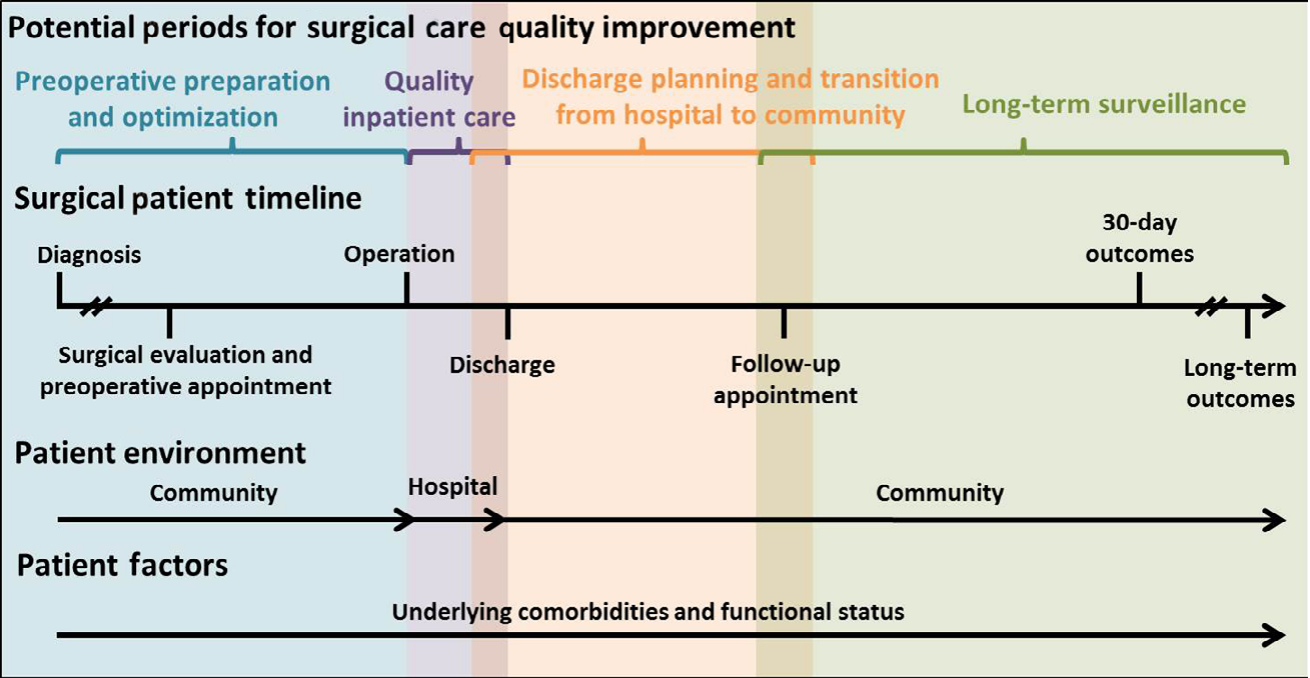
Inpatient surgical care pathway. Advance scheduling of most surgical admissions provides an opportunity to plan for post‐discharge care (including availability of informal caregivers), time for comprehensive risk assessment, and patient self‐management training during pre‐operative and inpatient periods.

Unlike most medical admissions, most surgical admissions are elective or scheduled in advance (Figure 1). In other words, the patient has some control over the timing of surgery. Potential advantages of an elective admission include the opportunity to plan for postdischarge care, the time to perform a comprehensive risk assessment as well as to provide training and education about self‐management to patients and family members. Hence, the conceptual model of surgical admission needs to account for patient and provider communication, shared decision‐making, risk assessment and patient and care‐giver training – factors that play a much smaller role in urgent medical admissions and readmissions. Whether patients and providers use these opportunities for preparation offered by elective surgical admissions is not known.

## Background

As the population ages, the numbers of older patients with cancer are likely to increase. If current patterns of cancer incidence hold, by 2030, 70% of persons with cancer will be over 50 years of age and over 65% of deaths will occur in this age group. Approximately, 400,000 older patients with cancer are currently alive in the USA and at least two‐thirds require management of their cancers and other comorbid conditions (Balducci & Stanta 2000). Burden of illness and complexity of treatment regimens make it challenging for older oncology patients to participate in research, which impedes researchers’ ability to develop and test effective intervention for this population group.

Another challenge of evaluating interventions for older cancer patients is the choice of the primary outcome. While most oncology clinical trials traditionally focused on reducing mortality and recurrence at any cost, there is also growing pressure from public payers and government to control cost of medical care and use of unnecessary services, such as unplanned readmissions and excessive inpatient days. Furthermore, because surgery may exacerbate comorbid problems, a crucial first step is to adequately assess patients with regard to comorbid illness, disability and geriatric syndromes. Once these steps are taken, evaluation of interventions to improve overall outcomes can focus not only on survival but also on maintenance of function and improvement of quality of life in the context of postoperative care.

Several studies have attempted to identify risk factors predictive of adverse perioperative outcomes in the elderly – for example, emergency surgery, American Society of Anesthesiologists’ perioperative risk score (ASA), pre‐operative comorbidities and advancing age – but the evidence is inconsistent and surgical decision algorithms remain unsatisfactory (Leung & Dzankic 2001). The discrepancy in data may in part reflect the lack of information concerning common risk factors unique to the elderly cancer population. Identification of pre‐operative markers reflecting the variability in cancer patients may help predict poor outcomes and aid in pre‐operative decision‐making. Current risk stratification models, such as Colorectal Physiologic and Operative Severity Score for enumeration or Mortality and Morbidity (CR‐POSSUM) (Tekkis *et al*. 2004, Bromage & Cunliffe 2007) and National Surgery Quality Improvement Program (NSQIP) morbidity and mortality risk calculator (Cohen *et al*. 2009), employ chronological age as a predictor of adverse perioperative outcomes; chronologic age, however, does not accurately reflect functional, physical and cognitive decline or socio‐economic barriers and availability of care‐giver support.

To date, efforts to improve postdischarge outcomes have focused on postdischarge care coordination and inpatient education for high‐risk medical patients, with mixed results (Dhalla *et al*. 2014, Leppin *et al*. 2014). Multidisciplinary multimodal programmes and interventions that focus on factors affecting poor outcomes (e.g. patient–provider communication, early postdischarge primary care physician (PCP) visit, care transition coaches, medication reconciliation and patient ability for self‐care) are more effective than single‐modality interventions. However, little is known about the effectiveness of care planning and the role of APPs on reducing surgical readmissions (Azimuddin *et al*. 2001, Kelly *et al*. 2013, 2014).

Since hospitals and health care providers will likely be held accountable for unplanned surgical readmissions and other surgical outcomes, it is imperative to learn how to recruit and retain surgical patients for research programmes focused on postdischarge care along with how to implement study protocols in a busy surgical clinic or unit. The focus on quality improvement and outcomes becomes even more important for all providers after CMS introduced several programmes giving hospitals a financial incentive to improve quality of care and share their quality metrics with the CMS and consumers (Cms.Gov 2013, 2014a).

## The study

### Study aim

The aim of this study was to explore the feasibility of recruiting surgical oncology patients and implementing a surgical integrated discharge (SID) programme led by advanced practice providers (APP). The purpose of the study was to examine the barriers and facilitators of patient recruitment and retention in an inpatient surgical setting and evaluate resources required to implement a surgical integrated discharge intervention. Finally, we explored the feasibility of using administrative and clinical institutional databases for data collection, to reduce the burden of data collection on patients.

#### Study design

**Figure 2:**
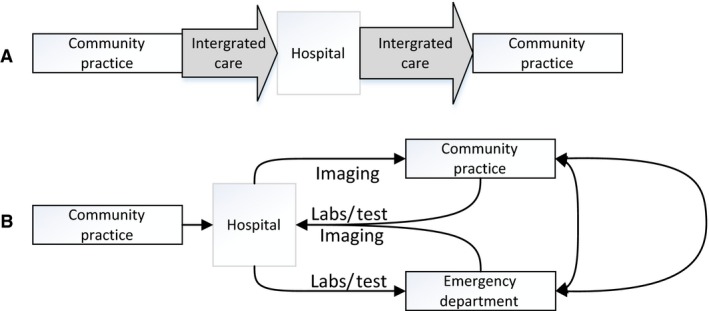
Study Flowchart. The integrated care approach (A) started as soon as the decision about surgery was made and focused on streamlining care, avoiding delays, and minimizing patient burden. Usual surgical care pathways (B) are characterized by unnecessary appointments, redundant tests, and care inefficiencies.

To develop the study protocol and guide the analysis and result presentation, we followed the commonly used Standards for Quality Improvement Reporting Excellence (SQUIRE 2.0) (Ogrinc *et al*. 2015). Because of the volume and organization of surgical services, no concurrent blinded two‐arm randomization design was achievable. Hence, we used a longitudinal prospective observational design. Each clinical service identified a lead inpatient APP, an outpatient APP and a lead attending surgeon to coordinate study recruitment and follow‐up. The lead APPs for each service worked together to coordinate care, patient training, discharge procedures and postdischarge follow‐up through weekly in‐person meetings involving all study APPs, shared electronic medical records and daily virtual communication. All patient care‐related issues and study protocol problems were addressed at weekly research team meetings which included the project staff and APPs (Figure 2).

**Figure 3:**
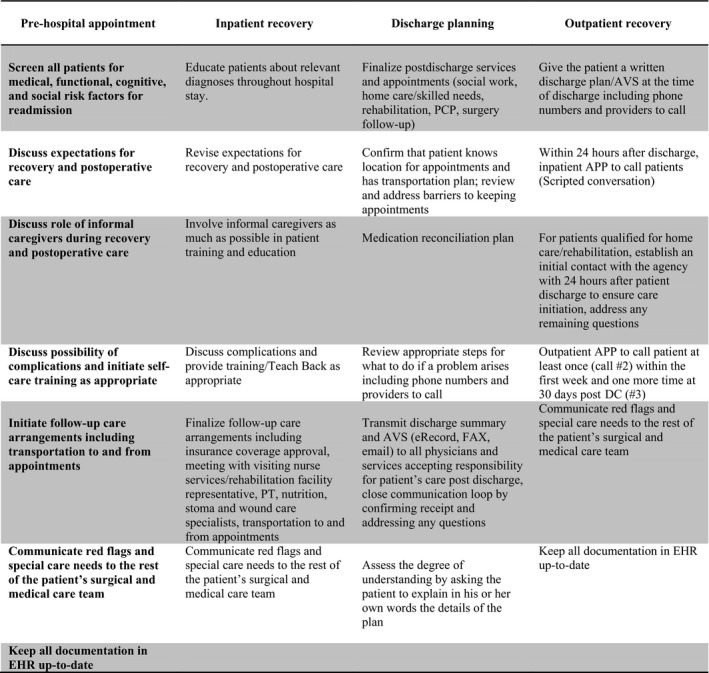
Summary of integrated care intervention. Each patient‐ and care‐related activity of this multidisciplinary intervention was incorporated into organization strategy and work flow diagrams for each care setting were modified appropriately.

The APPs served as patient gatekeepers and liaisons with other providers before, during and after surgical admission. At the time of discharge, patients were provided with phone numbers to contact the care team directly during regular business hours as well as after hours. Within 24 hours after discharge, the APP contacted the newly discharged patient with a personal call to ensure the patient was following discharge instructions such as taking medications including VTE prophylaxis and maintaining appropriate care of the stoma and other wounds. In addition, the APP confirmed that follow‐up appointments with the PCP and the surgeon had been scheduled, helped arrange patient transportation if needed, ensured that the visiting nurse service had initiated contact as appropriate and addressed any remaining postdischarge concerns (Figure 3).

#### Study participants

Between August 2013–October 2014, eligible patients were identified through daily screening of surgical clinic schedules. Patients aged 50 and older with colorectal, bladder or pancreatic cancer and being considered for surgery were eligible. After surgery, any patients who were discharged to another facility were excluded from the study.

A comprehensive physiologic, functional and social assessment of patients at the time of pre‐operative assessment was conducted to identify any patients with high unmet needs and those at risk for readmission, The intervention was first implemented for bladder cancer patients in August 2013, followed by colorectal (November 2013) and pancreatic cancer patients (January 2014).

### Comparison cohort

**Figure 4:**
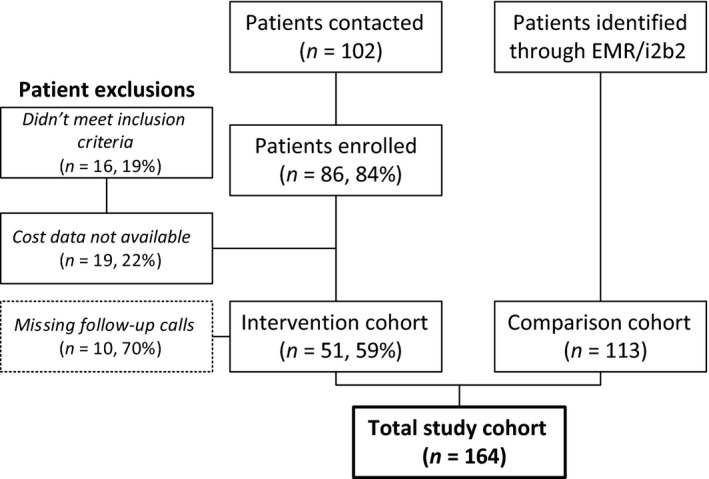
Study Enrollment. We recruited colorectal, bladder and pancreatic cancer patients age 50 and older who were considered for surgery between October 2013 and October 2014, and who were discharged home, with or without home care. Control group patients were selected using electronic medical records from surgical inpatients who underwent the same elective procedures a year prior to the intervention implementation (July 2012‐July 2013).

Control group patients were selected from the institutional electronic medical record system (EPIC) using the *‘*Informatics for Integrating Biology and the Bedside (i2b2)*’* electronic data system. Patients matched by demographic (age, gender, race) and clinical (tumour type, stage) characteristics (Figure 4), who underwent the same elective procedures as the study cohort (*‘*Cystectomy*’*,* ‘*Whipple*’*,* ‘*Colectomy*’*,* ‘*Ileocolostomy*’*,* ‘*Low anterior resection*’*,* ‘*Hemicolectomy*’*,* ‘*Sigmoidectomy*’*) in the year prior to the intervention (July 2012–July 2013) were selected for comparison. i2b2 is an NIH‐funded informatics framework that allows users to query existing clinical data (EPIC) and form patient cohorts for prospective follow‐up or retrospective analysis.

#### Description of programme implementation

The integrated care approach started as soon as the decision about surgery was made, at the first pre‐operative meeting between the patient and an APP (Figures 2 & 3). The intervention included a comprehensive pre‐operative patient assessment to identify any medical problems or unmet needs that would require management during the postoperative period, advance multidisciplinary communication between inpatient and outpatient surgical care teams (weekly multidisciplinary APP meetings, twice a week meetings between clinical and study teams), at least three postdischarge follow‐up phone calls with the patient (at 2‐3 days, 1‐2 weeks and 30 days postdischarge) and daily care coordination communication between the surgical team and advanced care partners (APPs: nurse practitioners, unit managers, care coordinators, visiting nurse services and social workers). The intervention also focused on improving patient and staff adherence to existing evidence‐based practice guidelines (e.g. prompt provider communication after patient appointments, comprehensive pre‐surgical patient assessment) and reducing existing system limitations (e.g. patient confusion about which number to call in case of emergency after discharge, proactively screening postdischarge patients for early symptoms of problems that could be addressed in the clinic, before the patient decides to go to the emergency room).

Three major amendments to the original study protocol were made a month after starting the enrolment. To minimize disruption for the clinic schedule, we changed the process of identifying new potential subjects. Instead of surgical attendings nominating eligible patients in their clinics, the study coordinator and outpatient APP prospectively reviewed each clinic's schedule every day. We also expanded the eligibility age from 70‐50 years of age after we identified early in the study several younger patients with multiple risk factors for readmission and complications. Finally, to reduce patient stress and improve study enrolment rate, we made every effort to schedule pre‐operative clinic visits early in the day and give patients detailed instructions ahead of time about how long the appointment was going to take so that they could have realistic expectations and schedule accordingly. This allowed study staff adequate time to conduct all the necessary assessments and forms and answer all questions patients may have about the study.

#### Recruitment feasibility and data quality

Among contacted patients, the enrolment rate was 84%. The main reason for refusal was patients’ perceived lack of time for baseline assessment. Overall, 10 patients had missing follow‐up phone calls, mainly the second and third follow‐up calls, which is equivalent to about a 7% missing call rate (Figure 4). One patient had two missing ASA values which were imputed. The two main reasons for missing follow‐up calls were patients not responding/not returning calls and the APP not having time to make a call. Twenty‐five patients of 86 (30%) did not have completed baseline evaluations, either because the patient did not return completed forms to the study coordinator or because the patient was admitted with less than 48‐hour notice.

#### Data analysis

Initially, 86 patients agreed to participate in the study and were followed up for up to 120 days after hospital discharge. Our final sample consisted of 164 patients, of whom 51 were in the case group and 113 were in the control group (Figure 4). After identifying patient cohorts, we abstracted data from electronic medical records and billing systems to obtain additional patient information (e.g. insurance status, comorbidities) and to calculate three outcomes: length of inpatient stay (LOS), 30‐day readmission rate and total 90‐day healthcare costs, including charges for inpatient and outpatient services.

The primary outcome in this study was patients’ LOS after surgery. Secondary outcomes included readmission rate and overall 90‐day healthcare costs since the day of admission. We controlled for other patient risk factors using Charlson comorbidity scores (Charlson *et al*. 1987), blood loss volume during the procedure, ASA scores, race, gender and age. Two dummy variables, *‘*pancreatic*’* and *‘*bladder*’* were created to compare patients by tumour type. We also included a continuous time variable to account for any institutional or policy changes affecting inpatient services between 1 January 2012 and the date of each patient's admission, as well as any time–intervention interaction.

To compare the intervention and control groups, we used the *t*‐test or Wilcoxon test for continuous variables and chi‐squared or Fisher's exact test for categorical variables. We performed bivariate analysis to explore the relationship between patient characteristics and outcomes (LOS, 30‐day readmissions rate, 90‐day total costs). We then developed a multivariate risk‐adjusted model to estimate the effect of the intervention on the outcomes, controlling for patient risk factors, which could independently affect the outcomes (e.g. patient demographics and comorbidity). The LOS was modelled using a negative binomial model to remedy for overdispersion (Abdul‐Aziz *et al*. 2013). Readmission rate was modelled using a logistic regression model. For the cost analysis, we used a multivariable linear regression model on log‐transformed total costs.

### Validity and reliability

As this was a feasibility study, we used an adaptive design allowing for changes in enrolment criteria without interim analysis of the treatment effect (blinded adaptation). All analyses were performed using SAS 9.4 (SAS Institute, Cary, NC, USA). We used the Markov Chain Monte Carlo (MCMC) multiple imputation method to impute missing values (i.e. ASA scores) (Patterson 2007).

#### Ethical considerations

The study was approved by the University of Rochester Institutional Review Board (Study ID00038826). With the attending approval, an advanced practice provider (APP) introduced the study to the patient after the pre‐operative assessment appointment. Interested patients then met with the study coordinator to obtain more information about the study and to complete the consent process. No pressure was applied to any person to participate and we were especially considerate to patients and caregivers who were unable to stay for the enrolment evaluation or unavailable to take a follow‐up phone calls by offering alternative times.

## Results

### Description of participants

Overall, 86 patients and 113 controls were identified (Figure 4, Table 1). The intervention and control cohorts were similar at baseline (about two‐thirds male, 40% colorectal cancer, 40% bladder cancer, 20% pancreatic cancer, over 90% Whites, on average 2·7 comorbidities, ASA = 2·8) except for age. Patients in the intervention group were older than control patients (72 vs. 67 years old, range 50‐90). The baseline readmission rate was 17%, ranging from 8% among colorectal patients to 23% among patients with bladder cancer.

**Table 1:** Patients characteristics

	Cases (*N = *51)	Controls (*N = *113)	*P* value
Race			
Black	2 (3·92)	5 (4·42)	0·910
Other	2 (3·92)	7 (6·19)	
White	47 (92·16)	101 (89·38)	
Sex			
Female	21 (41·18)	39 (34·51)	0·412
Male	30 (58·82)	74 (65·49)	
Cancer type			
Colorectal	22 (43·14)	41 (36·28)	0·664
Pancreatic	9 (17·65)	25 (22·12)	
Urology	20 (39·22)	47 (41·59)	
Insurance			
Medicare	27 (52·94)	51 (245·13)	0·640
Others	2 (3·92)	6 (5·31)	
Private	22 (43·14)	56 (49·56)	
Readmitted			
Not Readmitted	42 (84·31)	94 (83·19)	0·857
Readmitted	8 (15·69)	19 (16·81)	
Age	72·02 (8·39)	66·88 (9·49)	0·001
Comorbidity score	2·69 (2·09)	2·66 (1·8)	0·944
ASA score	2·84 (0·58)	2.8 (0·6)	0·642
Time	742·98 (198·91)	478·42 (102·9)	<0·001
Total costs	26607 (17220)	22827 (14669)	0·150
Length of stay	8·78 (6·95)	8·02 (5·81)	0·463

ASA Score, American Society of Anesthesiologists’ perioperative risk score; LOS, length of stay.

### Postsurgical and postdischarge outcomes

Among enrolled patients in the intervention arm, 59% (*n* = 51) had non‐missing data adequate for the analysis, with similar missing data rate among controls (62%, *n* = 113). Most missing data were comorbidity status (obtained from electronic health records using i2b2) and charges (from billing system). In the intervention arm, five patients stopped returning phone calls (10 missed phone calls total). Completion of baseline assessment varied widely, from 100% for in‐office frailty test to less than 5% (*n* = 2) for exercise physiology assessment.

On univariate analysis, LOS did not differ between the intervention and control cohorts (8·0 vs. 8·8 days, *P* = 0·5). After controlling for patient demographics, as shown in Table 3 using a negative binomial regression model, clinical characteristics and time trends, integrated care was found to be associated with shorter LOS (IRR = 0·42, *P* = 0·034). There was no significant difference in the readmission rates between the intervention and control groups on both bivariate (15·7% vs. 16·8%, *P *= 0·9) and multivariate analyses (OR = 0·94, *P* = 0·9). The overall costs tended to be higher in the intervention arm (univariate analysis: $26,607 vs. $22,827, P = 0·15), with the difference reaching significance in the multivariate analysis of log‐transformed costs (IRR = 1·15, *P* = 0·028).

Our results demonstrated significant differences in enrolment, implementation and adherence to the intervention by cancer type (surgical unit), risk factors and demographics. The highest rate of enrolment and programme completion was among bladder cancer patients. After adjusting for differences in patient demographics and disease severity, patients with colorectal cancer had significantly lower average LOS, both with and without the intervention (7·7 vs. 8·0 days for bladder cancer and 9·9 days for pancreatic cancer). CRC patients also had lower risk‐adjusted 90‐day costs (median for colorectal $21,085, bladder $26,399, pancreatic $24,684, *P* < 0·001) (Table 2). Older age and greater amounts of perioperative blood loss were consistently associated with longer LOS and higher costs of 90‐day care episodes (Table 3).

**Table 2:** Univariate analyses of the study outcomes: length of stay, readmissions and total 90‐day total healthcare costs, by individual risk factors

Name	Description	LOS, Mean days	Not readmitted *N* (%) sd *n = *137		Readmitted *N* (%) sd *n = *27		Total Cost Mean $
Race	Black	7·14	7 (5·11)		0 (0)		21,046
	Other	7·11	8 (5·84)		1 (3·7)		21,618
	White	8·38	122 (89·05)		26 (96·3)		24,287
Sex	Female	7·87	53 (38·69)		7 (25·93)		22,822
	Male	8·48	84 (61·31)		20 (74·07)		24,683
Cancer type*	Colorectal	7·68	58 (42·34)		5 (18·52)		21,085
	Pancreatic	9·88	27 (19·71)		7 (25·93)		24,684
	Bladder	7·97	52 (37·96)		15 (55·56)		26,399
Insurance	Medicare	9·14	64 (46·71)		14 (17·95)		26,968
	Others	7·38	7 (5·11)		1 (3·7)		23,701
	Private	7·46	66 (48·18)		12 (15·38)		21,067
Intervention	Case	8·78	43 (31·39)		8 (29·63)		26,607
	Control	8·02	94 (68·61)		19 (70·37)		22,827
Age			68·34	9·41	69·15	9·74	
Comorbidity			2·66	1·96	2·7	1·46	
ASA Score			2·82	0·61	2·78	0·51	
Follow‐up, days			550	183	580	170	

The *P* values were calculated by anova for numerical covariates; and by chi‐square test or Fisher's exact for categorical covariates (*P < 0·05).

ASA score, American Society of Anesthesiologists’ perioperative risk score; LOS, length of stay.

**Table 3:** Multivariate analysis of the impact of patient and tumour characteristics on inpatient length of stay, readmission rate and total 90‐day costs

Variable	Total cost	Length of stay	Readmission
	Coefficient	IRR	OR
	0·01^a^	1·01^a^	1·01
Black vs. White	−0·13	1·05	<0·001
Other vs. White	0·18	0·85	0·49
Male vs. female	0·01	1·00	1·30
Pancreatic cancer	0·24^a^	1·25	2·86
Bladder cancer	0·08	0·75^a^	2·92
Blood Lost	0·19^a^	1·23^a^	
Case vs. Control	0·37^a^	0·42^a^	0·94
ASA Score		1·08	
Comorbidity Score		1·01	
Follow‐up, days		1·00^a^	

a *P* < 0·05.

ASA Score, American Society of Anesthesiologists’ perioperative risk score; IRR, incidence rate ratio from negative binomial model (IRR >1 means greater LOS compared with the reference group); OR, odds ratio from logistic analysis (OR >1 means greater readmission risk).

## Discussion

### Discussion of results

Our multimodal SID programme included several tightly linked components, including pre, peri‐ and postsurgical care coordination and enhanced communication approaches. We demonstrated that implementation of this advanced surgical discharge planning programme was associated with shorter hospital LOS (reducing LOS on average by 1 day), without any negative effect on readmission rates. Our analysis also indicated that the healthcare costs in the intervention arm (after the integrated intervention protocol was implemented) were higher than costs during the baseline (preceding) year for the patients in the control arm. Our findings also demonstrated the importance of an institutional culture of quality improvement including clear expectations, standardized protocols and staff buy‐in and training.

Several reasons may explain why costs were higher in the intervention group compared with the controls. First, the hospital billing data may not reflect the efficiency gains (e.g. in the use of medications, in‐hospital tests and procedures and staff time on activities included within the 90‐day surgical global fee) associated with the integrated care approach. It is also conceivable that extensive follow‐up and postdischarge communication with patients resulted in more frequent outpatient non‐surgical primary care and specialist visits within the first 90 days after discharge, not included in the 90‐day surgical global fee, which helped offset some of the future healthcare cost reductions not captured here (due to fewer long‐term complications, earlier diagnosis of cancer recurrence and slower functional decline) (Nicolaije *et al*. 2015). Some medications associated with the protocols may have increased the immediate costs (for instance, use of alvimopan to reduce side effects of narcotic medicines used to control postsurgical pain in patient who underwent gastrointestinal surgery). Alternatively, the higher costs may have resulted from annual changes in payer contracts and reimbursement schedules, unrelated to the intervention. In addition, robotic surgery, which was used in both groups but more often in the intervention group, has higher direct medical costs. Last, the higher intervention costs may reflect the non‐randomized study design; a small group of patients in the later time period with outlier prolonged lengths of stay and complications may have occurred by chance alone. This issue needs to be further addressed with a randomized, concurrently controlled, multi‐institutional study design.

The results of our study are consistent with recent reports of other similar interventions (Dhalla *et al*. 2014, Leppin *et al*. 2014). Among studies testing readmission reduction interventions, studies published before 2002 demonstrated greater effect (RR, 0·56 [95% CI, 0·40‐0·79]). This probably has to do with the fact that even simple system changes, like implementation of a new care coordination checklist, require time to penetrate the system and for staff to learn, practice and master (Johnson *et al*. 2004, Sartorius 2006). Hence, complex interventions that require participant learning may require a longer evaluation period to allow for intervention maturity and to fully capture the intervention effect. Furthermore, the standards of care have improved over time, biasing the intervention effect towards the null (Dhalla *et al*. 2014, Leppin *et al*. 2014).

In addition to integrated care model tested here, other strategies for preventing avoidable harm and minimizing the risk of adverse events in surgical patients are also available. These include implementation of standardized evidence‐based care pathways, such as the enhanced surgical recovery pathway (Arriaga *et al*. 2009, Varadhan *et al*. 2010) and escalation of care pathways (EOC). The aim of the enhanced recovery after surgery (ERAS) pathway, or *‘*multimodal rehabilitation*’*, is to attenuate the stress response to surgery and enable rapid recovery. The ERAS pathway may include pre‐operative counselling, no bowel preparation, no premedication, no pre‐operative fasting, provision of clear carbohydrate enriched liquids until 2 hours before surgery, non‐opioid analgesia, early removal of bladder catheters and early postoperative feeding and mobilization, among others. The ERAS protocol has been demonstrated to reduce LOS and complications, but it has no effect on mortality or readmissions (Arriaga *et al*. 2009, Varadhan *et al*. 2010) (Wick *et al*. 2015). The EOC involves recognition of patient deterioration and timely communication of this information to a senior colleague who could then arrange definitive management. The concept of EOC has been based on safety procedures developed for high‐risk industries like aviation, auto manufacturing and the military where redundancy mechanisms are incorporated into the system to compensate for a potential failure at any one point – for example, through the use of backup behaviours, dual‐tasking and debriefing (Catchpole *et al*. 2010, Johnston *et al*. 2015). Future studies may learn from ERAS and EOC techniques and incorporate the most relevant approaches into surgical readmission reduction programmes.

On the health system level, the main challenges for the successful implementation of the intervention were lack of integration between the initiative and other organizational changes and lack of organized and consistent training for involved clinical and administrative personnel (Salas *et al*. 2008, Salas & Rosen 2013). When the intervention was implemented as a department‐wide quality improvement programme as in the Department of Urology in our study, including strong leadership support and emphasis on departmental culture change, we observed greater provider adherence and less variation in their performance, similar to other published reports (Henrickson *et al*. 2009, Hicks *et al*. 2014). Other barriers to implementation included misaligned financial and clinical incentive, lack of care coordination and limited patient self‐care ability (Lucas & Pawlik 2014, Barnett *et al*. 2015).

### Study limitations

As this was a feasibility study, we focused on the assessment of processes and resources, not the effect size. Hence, our quantitative findings should be interpreted with caution, mainly to illustrate feasibility of collecting the necessary data. We also did not control for temporal trends due rolling enrolment, study personnel and clinicians’ learning curve, or seasonal fluctuations in‐hospital census which may have resulted in biased estimates of costs and rates. Similarly, we were unable to control for statistical variation in processes of care among different hospital units (e.g. APP scope of work, enhanced recovery protocols and after‐hours call triage policies) (Aiken *et al*. 2002, Kane *et al*. 2007, Catchpole *et al*. 2010, Petrovic *et al*. 2012, Zhu *et al*. 2012). Finally, several surgical attendings implemented some elements of the ERAS protocol during the study control period (July 2012–July 2013), which may have biased the comparison results towards the null.

In summary, this study demonstrates the feasibility of developing and implementing a surgery‐specific integrated care coordination programme using a multidisciplinary multimodal collaborative approach, recruiting surgical oncology patients undergoing major surgery for their cancer and the importance of the APPs role in surgical care integration. To improve patient short‐term (in‐hospital) and long‐term (postdischarge) outcomes, the implementation of such programme will require addressing current barriers to effective pre‐, peri‐ and postoperative care coordination; communication among patients, informal caregivers and healthcare teams and management of patient expectations and unmet needs. Further research is needed to understand patient preferences for engagement into integrated surgical care pathways, determine optimal times and settings for the integrated care processes and define the minimum necessary intervention *‘*core*’* components to guide financially sustainable practice changes. We conclude that strong institutional support, provider collaboration across various care settings and alignment of financial and clinical incentives are the key elements of successful implementation and dissemination of integrated surgical readmission reduction programmes.

## Author contributions

Conception and design – KN, JBK, LS, EG, FF, JRTM, TF, SM, EM; Acquisition of data – KN, JBK, SM, TF; Analysis and interpretation of data – KN, JBK, WP, XC, SM, TF; Drafting and revising the article critically for important intellectual content – KN, JBK, LS, EG, FF, JRTM, TF, SM, EM; Final approval of the version to be published – KN, JBK, LS, EG, FF, JRTM, WP, XC, TF, SM, EM.

All authors have agreed on the final version and meet at least one of the following criteria [recommended by the ICMJE (http://www.icmje.org/recommendations/)]:

- substantial contributions to conception and design, acquisition of data or analysis and interpretation of data;
- drafting the article or revising it critically for important intellectual content.
